# Conservation and Enhanced Binding of SARS-CoV-2 Omicron Spike Protein to Coreceptor Neuropilin-1 Predicted by Docking Analysis

**DOI:** 10.3390/idr14020029

**Published:** 2022-03-29

**Authors:** Piyush Baindara, Dinata Roy, Santi M. Mandal, Adam G. Schrum

**Affiliations:** 1 Departments of Molecular Microbiology & Immunology, Surgery, and Biomedical, Biological, & Chemical Engineering, School of Medicine, College of Engineering, University of Missouri, Columbia, MO 65211, USA; schruma@health.missouri.edu; 2 Department of Zoology, Mizoram University, Aizawl 796004, Mizoram, India; sdinaroym9@gmail.com; 3 Central Research Facility, Indian Institute of Technology Kharagpur, Kharagpur 721302, West Bengal, India; mandalsm@gmail.com

**Keywords:** SARS-CoV-2, Omicron, spike protein, neuropilin-1, CendR, COVID-19

## Abstract

The Omicron variant of SARS-CoV-2 bears peptide sequence alterations that correlate with a higher infectivity than was observed in the original SARS-CoV-2 isolated from Wuhan, China. We analyzed the CendR motif of spike protein and performed in silico molecular docking with neuropilin-1 (Nrp1), a receptor–ligand interaction known to support infection by the original variant. Our analysis predicts conserved and slightly increased energetic favorability of binding for Omicron CendR:Nrp1. We propose that the viral spike:Nrp1 coreceptor pathway may contribute to the infectivity of the Omicron variant of SARS-CoV-2.

## 1. Introduction

In December 2019, the first case of severe acute respiratory syndrome coronavirus 2 (SARS-CoV-2) was identified in Wuhan, China [1]. Within a few months, the World Health Organization (WHO) declared a global Coronavirus Disease 2019 (COVID-19) pandemic [2]. SARS-CoV-2 has been continuously evolving, and multiple variants have been reported, which are categorized as variant being monitored (VBM), variant of interest (VOI), or variant of concern (VOC) [3]. An early variant of SARS-CoV-2 was found to bear a D614G mutation in the spike glycoprotein, and it was associated with a high transmission rate that facilitated its global dispersion [4]. Up to the present, five main variants of concern have been identified, including Alpha (B.1.1.7), Beta (B.1.351), Gamma (P.1), Delta (B.1.617.2), and Omicron (B.1.1.529), all of which have been associated with high infectivity and transmission [5,6]. These properties are largely driven by the ability of viral mutations to (i) mediate evasion of immune mechanisms including neutralizing antibodies [7] and (ii) enhance the fitness of virus–host interactions that favor viral proliferation and dissemination [8]. Regarding the Omicron variant, three sublineages have been identified and named BA.1, BA.2, and BA.3, with BA.1 undergoing the greatest global dissemination to date [9], and BA.2 cases currently rising [10].

The present work focuses on a SARS-CoV-2 coreceptor pathway as a mechanism by which sequence variations may affect virus–host interactions. Largely, much of the analysis of major sequence alterations between different variants has emphasized SARS-CoV-2 spike glycoprotein [11], where the receptor binding domain (RBD) and amino (N)-terminal domain (NTD) are particularly relevant to infectivity [7]. RBD provides a primary site of contact via the receptor ACE2 on the host cell, an interaction that is central to infection by all described variants, including Omicron [12,13]. Recently, Nrp1 was identified as a host coreceptor for SARS-CoV-2 infection [14]. Nrp1 is a cell surface receptor that regulates pleiotropic biological processes and can play important roles in angiogenesis, vascular permeability, and development of the nervous system [15]. It is known that Nrp1 can interact with several proteins having a C-end rule (CendR) sequence, represented by a polybasic sequence motif, R/KX XR/K, located at the C-terminus [15,16]. In SARS-CoV-2 spike glycoprotein, the polybasic sequence motif Arg-Arg-Ala-Arg (RRAR) provides a cleavage site for host furin protease activity that results in two polypeptides: S1 and S2. It has been shown that Nrp1 can bind to the S1 polypeptide via its CendR motif, facilitatating viral entry and infectivity in COVID-19 [14]. Together, maximum infection potentiation was observed when Nrp1 and ACE2 were co-expressed on the same cell types in the same tissues [17].

Because of the high infectivity and transmissibility demonstrated for the Omicron variant [18], we analyzed sequence alterations in the polybasic sequence motif and performed in silico molecular docking experiments between Nrp1 and the CendR motif of spike protein, comparing results with those of the original SARS-CoV-2 variant isolated from Wuhan. Together with tissue proteomic co-expression analysis, the data support a model where conserved and possibly enhanced Nrp1:CendR interaction may contribute to Omicron variant infectivity and transmissibility.

## 2. Materials and Methods

### 2.1. Protein Retrieval and Preparation

The spike protein sequence of Omicron variant (B.1.1.529) was retrieved from GISAID database (EPI_ISL_6752027), while the spike protein sequences of Wuhan variant (Wuhan-Hu-1) and Delta variant (B.1.617.2) were retrieved from NCBI database (Ref seq: YP_009724390.1 and Gene bank: QWK65230.1, respectively). The human receptor of SARS-CoV-2, Nrp1 (PDB ID: 2QQM; (http://www.rcsb.org (accessed on 15 January 2022)) was used for in silico docking experiments with CendR of Omicron or the original variant isolated from Wuhan. Target protein and complex structure modifications were carried out by removing ligands, ions, and/or water molecules by using Chimera 1.15 (https://www.cgl.ucsf.edu/chimera/ (accessed on 15 January 2022)).

### 2.2. Homology Modeling and Sequence Alignment

CendR motifs of both Omicron and the original variant isolated from Wuhan were prepared by using the Swiss model (https://swissmodel.expasy.org/ (accessed on 15 January 2022)) online platform. Further refinements of motif structures and amino acid sequence alignment were performed by using Discovery Studio Visualization [19] and bioedit (http://www.mbio.ncsu.edu/bioedit/bioedit.html (accessed on 15 January 2022)), respectively.

### 2.3. Molecular Docking

Molecular docking experiments were performed in silico by using HDOCK online server: http://hdock.phys.hust.edu.cn/ (accessed on 15 January 2022) [20]. The best protein–protein complex was selected from the top ten conformers based on docking score (free binding energy, kcal/mol). The interaction analysis was conducted using PyMOL [21] and Discovery Studio Visualization [19] software packages.

### 2.4. Protein Expression Tissue Profiling

Protein expression profiles of human ACE2 (ENSG00000130234) and Nrp1 (ENSG00000099250) were visualized and acquired from The Human Protein Atlas (https://www.proteinatlas.org/ (accessed on 16 January 2022)).

## 3. Results and Discussion

### 3.1. Predicting Nrp1 Coreceptor Compatibility for the Omicron Variant of SARS-CoV-2

**Figure 1:**
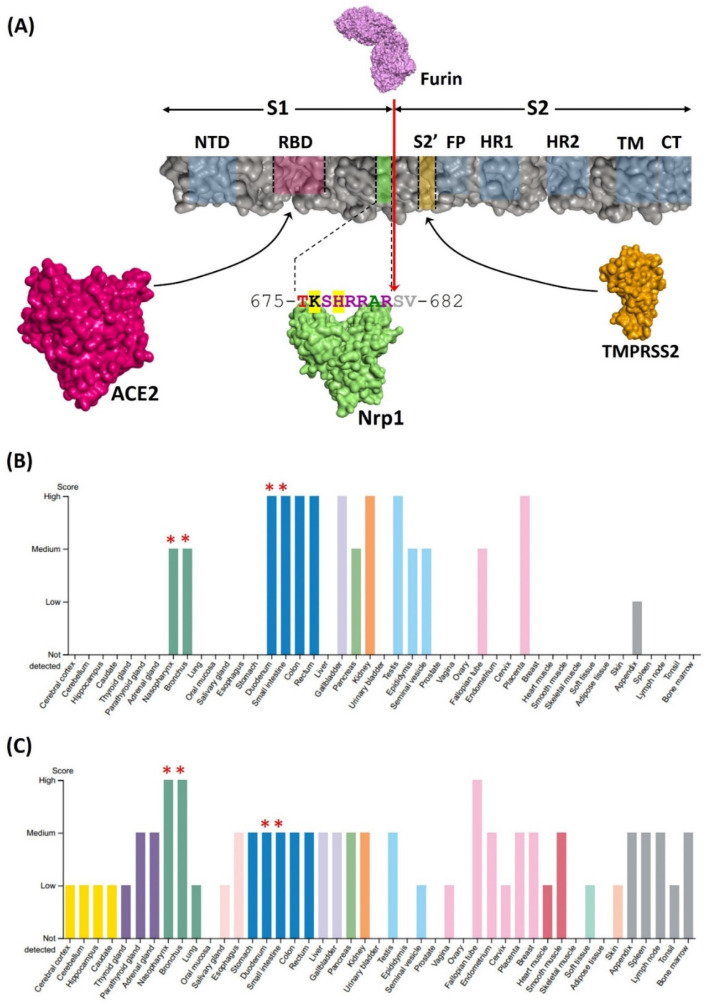
NRP1 as a coreceptor for SARS-CoV-2 infection. (**A**) ACE2, Nrp1, and TMPRSS2 proteins and color code-matched binding sites on spike protein domains are illustrated. The amino acid sequence of the CendR motif of the S1 domain of spike protein is shown for Omicron, where sequences altered relative to the original Wuhan variant are highlighted in yellow. The red arrowhead indicates the furin protease cleavage site that generates S1 and S2. NTD: N-terminal domain; RBD: Receptor Binding Domain; HR1 and HR2: Heptad repeats; TM: Transmembrane domain; CT: Cytoplasmic domain. (**B**,**C**) Data from Human Protein Atlas display tissue expression of (**B**) ACE2 and (**C**) Nrp1. Red asterisks (*) represent major infection sites of SARS-CoV-2, where both ACE2 and Nrp1 are co-expressed.

Despite published literature suggesting that Omicron may display increased infectivity over the original Wuhan variant, previous comparative studies between ACE2 and SARS-CoV-2 spike proteins did not reveal a substantial if any increase in binding affinity for Omicron compared to the other major variants [22,23,24]. The propensity of Omicron to evade immune mechanisms including neutralizing antibodies elicited by vaccination or prior exposure to other SARS-CoV-2 variants likely contributes to its enhanced infectivity and dissemination [7]. However, it is also possible that other variations in viral sequences could play a role. A model for spike protein domains, protease-mediated processing, and binding partners is presented where a furin protease cleavage site seperates a polybasic RRAR sequence present in the CendR motif of S1 that can bind Nrp1 (Figure 1A). Data from Human Protein Atlas confirmed that the tissue co-expression of ACE2 and Nrp1 was prominent at known SARS-CoV-2 infection sites, including nasopharynx, bronchus, and intestine (Figure 1B,C), where increased Nrp1 expression has been reported in COVID-19 patients [25]. Thus, known characteristics of spike protein processing, together with co-expression of both Nrp1 and ACE2, confirmed the availability of Nrp1 for possible coreceptor function in infection.

**Figure 2:**
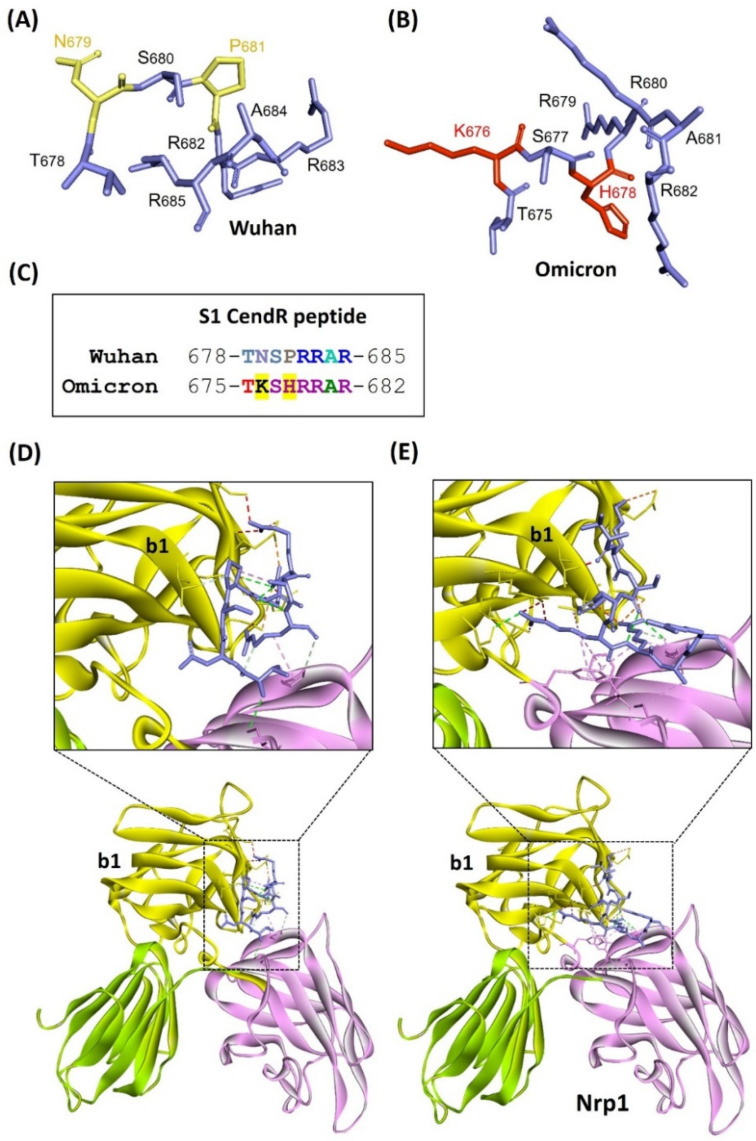
Homology modeling, sequence analysis for variant amino acids, and molecular docking analysis of CendR motif of original Wuhan isolate or Omicron variant with Nrp1. (**A**) CendR motif of original Wuhan isolate of SARS-CoV-2. (**B**) CendR motif of Omicron with amino acids that are altered with respect to the original Wuhan variant shown in red. (**C**) Sequence alignment of CendR motifs from the original Wuhan isolate versus Omicron variant with amino acid alterations highlighted in yellow. (**D**) Nrp1 docking with CendR motif of original Wuhan isolate. (**E**) Nrp1 docking with CendR motif of Omicron variant. Interactions are shown in the zoomed image while the b1 domain (yellow color) contains the interaction site for the CendR motifs.

Therefore, we analyzed sequence alterations at the CendR binding site for Nrp1 of Omicron to generate a prediction of the potential for this variant to possibly maintain coreceptor activity. Omicron spike glycoprotein was observed to have 37 mutations when compared to the spike glycoprotein of the original variant isolated from Wuhan, with the highest number of mutations, 15, found in the RBD motif. We analyzed the CendR sequence motif of S1 protein for Omicron, Delta, and the original variant isolated from Wuhan. For Omicron and the original isolate from Wuhan, the polybasic sequence motif was mapped and the CendR binding site was aligned and displayed (Figure 2A,B). Notably, two sequence substitutions in Omicron (N679K and P681H) added basic amino acids lysine and histidine to the CendR motif (Figure 2C), which enhanced the overall basicity of the polybasic sequence of the Omicron variant. Furthermore, we performed in silico molecular docking between Nrp1 and CendR sequences of the Omicron variant and the original isolate from Wuhan to predict free binding energies. The free binding energy calculated between Nrp1 and the CendR sequence of the original variant from Wuhan (−180.40) provided a basis for comparison to the data for the Omicron variant. We found that free binding energy between the Omicron CendR sequence and Nrp1 (−208.99 kcal/mol) indicated a somewhat increased energetic favoribility of interaction (Table 1). Moreover, CendR sequences of both Omicron and the original variant from Wuhan were predicted to be involved in various interactions at the Nrp1 binding site (Figure 2D,E; Table S1). In contrast, comparing the original Wuhan variant to the Delta variant, Delta had only one amino acid alteration in CendR, with in silico docking results predicting a free binding energy that was almost unchanged (−180.49 kcal/mol) from that of the original Wuhan variant (Table 1).

These results largely support the prediction that the Nrp1 coreceptor mechanism could be conserved or possibly enhanced in the Omicron variant. Nrp1 is known to regulate the internalization of CendR ligands through an endocytic process like macropinocytosis, and Nrp1 is also involved in enhancing host infection in the case of Epstein–Barr virus [26,27]. It was shown that the Nrp1, b1 domain could directly bind to the S1 CendR sequence of SARS-CoV-2 and facilitate viral infection [14,17]. The present results showed the possible involvement of sequence alterations at the CendR sequence of the Omicron variant where in silico docking analysis predicted an increased energetic favoribility compared to the original isolate from Wuhan. This supports the possibility that the Nrp1 coreceptor pathway may contribute to the infectivity of the Omicron variant. The predicted conservation of this coreceptor pathway across several different variants of concern supports previously published suggestions that Nrp1 could represent a potential therapeutic target to combat SARS-CoV-2 infection [28,29,30].

### 3.2. Limitations of the Study

First, the present study is based on in silico analysis using molecular docking and structural aspects of Nrp1 and SARS-CoV-2 spike protein CendR from different variants. Empirical determination of binding affinities and detailed in vitro and in vivo studies will be required to test the predictions made here. Second, this study is limited to viral binding sites and proteins for which sequences and structures are known and publicly available. Third, protein expression data in this study rely on data archived and publicly available from The Human Protein Atlas. Despite these limitations, the present study provided some important predictions for molecular interactions that may affect infectivity characteristics of the Omicron variant. These predictions could be used or considered for the development of better treatment strategies and therapeutic drug development to mitigate the COVID-19 pandemic.

## 4. Concluding Remarks

This study calls attention to Nrp1 as a coreceptor for SARS-CoV-2 viral entry into the cell, predicting conserved and slightly increased energetic favorability of binding for Omicron CendR:Nrp1. We speculate that the viral spike:Nrp1 coreceptor pathway may contribute to the infectivity of the Omicron variant of SARS-CoV-2, supporting the suggestion of Nrp1 as a potential therapeutic target for the development of future antivirals against evolving SARS-CoV-2 variants. Overall, using the predictions from the present study, further detailed studies will be required for better management and development of therapeutic strategies to combat the COVID-19 pandemic.

## Figures and Tables

**Table 1:** Determination of free binding energy (kcal/mol) from the docking analysis of Omicron, Delta, and Wuhan variant CendR with Nrp1.

	Free Binding Energy (kcal/mol)		
CendR	Wuhan	Delta	Omicron
Nrp1	−180.40	−180.49	−208.99

## Data Availability

All data are included in the manuscript and Supplementary Materials, and further details are available upon request.

## Supplementary Materials

The following supporting information can be downloaded at: https://www.mdpi.com/article/10.3390/idr14020029/s1, Table S1: Amino acid interation analysis of docking complex between Nrp1 and S protein (CendR) of Wuahan and Omicron variant.

## References

[1] Worobey M. (2021). Dissecting the early COVID-19 cases in Wuhan. Science.

[2] Cucinotta D., Vanelli M. (2020). WHO declares COVID-19 a pandemic. Acta Biomed..

[3] Konings F., Perkins M.D., Kuhn J.H., Pallen M.J., Alm E.J., Archer B.N., Barakat A., Bedford T., Bhiman J.N., Caly L. (2021). SARS-CoV-2 Variants of Interest and Concern naming scheme conducive for global discourse. Nat. Microbiol..

[4] Volz E., Hill V., McCrone J.T., Price A., Jorgensen D., O’Toole Á., Southgate J., Johnson R., Jackson B., Nascimento F.F. (2021). Evaluating the Effects of SARS-CoV-2 Spike Mutation D614G on Transmissibility and Pathogenicity. Cell.

[5] Campbell F., Archer B., Laurenson-Schafer H., Jinnai Y., Konings F., Batra N., Pavlin B., Vandemaele K., Van Kerkhove M.D., Jombart T. (2021). Increased transmissibility and global spread of SARSCoV- 2 variants of concern as at June 2021. Eurosurveillance.

[6] Chen R.E., Zhang X., Case J.B., Winkler E.S., Liu Y., VanBlargan L.A., Liu J., Errico J.M., Xie X., Suryadevara N. (2021). Resistance of SARS-CoV-2 variants to neutralization by monoclonal and serum-derived polyclonal antibodies. Nat. Med..

[7] Harvey W.T., Carabelli A.M., Jackson B., Gupta R.K., Thomson E.C., Harrison E.M., Ludden C., Reeve R., Rambaut A., Peacock S.J. (2021). SARS-CoV-2 variants, spike mutations and immune escape. Nat. Rev. Microbiol..

[8] Plante J.A., Liu Y., Liu J., Xia H., Johnson B.A., Lokugamage K.G., Zhang X., Muruato A.E., Zou J., Fontes-Garfias C.R. (2021). Spike mutation D614G alters SARS-CoV-2 fitness. Nature.

[9] Takashita E., Kinoshita N., Yamayoshi S., Sakai-Tagawa Y., Fujisaki S., Ito M., Iwatsuki-Horimoto K., Halfmann P., Watanabe S., Maeda K. (2022). Efficacy of Antiviral Agents against the SARS-CoV-2 Omicron Subvariant BA.2. N. Engl. J. Med..

[10] Callaway E. (2022). Why does the Omicron sub-variant spread faster than the original?. Nature.

[11] Thakur V., Bhola S., Thakur P., Patel S.K.S., Kulshrestha S., Ratho R.K., Kumar P. (2021). Waves and variants of SARS-CoV-2: Understanding the causes and effect of the COVID-19 catastrophe. Infection.

[12] Miller N.L., Clark T., Raman R., Sasisekharan R. (2021). Insights on the mutational landscape of the SARS-CoV-2 Omicron variant. BioRxiv Prepr. Serv. Biol..

[13] Shang J., Wan Y., Luo C., Ye G., Geng Q., Auerbach A., Li F. (2020). Cell entry mechanisms of SARS-CoV-2. Proc. Natl. Acad. Sci. USA.

[14] Daly J.L., Simonetti B., Klein K., Chen K.E., Williamson M.K., Antón-Plágaro C., Shoemark D.K., Simón-Gracia L., Bauer M., Hollandi R. (2020). Neuropilin-1 is a host factor for SARS-CoV-2 infection. Science.

[15] Guo H.F., Vander Kooi C.W. (2015). Neuropilin functions as an essential cell surface receptor. J. Biol. Chem..

[16] Teesalu T., Sugahara K.N., Kotamraju V.R., Ruoslahti E. (2009). C-end rule peptides mediate neuropilin-1-dependent cell, vascular, and tissue penetration. Proc. Natl. Acad. Sci. USA.

[17] Cantuti-Castelvetri L., Ojha R., Pedro L.D., Djannatian M., Franz J., Kuivanen S., van der Meer F., Kallio K., Kaya T., Anastasina M. (2020). Neuropilin-1 facilitates SARS-CoV-2 cell entry and infectivity. Science.

[18] Chen J., Wang R., Gilby N.B., Wei G.-W. (2022). Omicron Variant (B.1.1.529): Infectivity, Vaccine Breakthrough, and Antibody Resistance. J. Chem. Inf. Model..

[19] Accelrys Software Inc. (2012). Discovery Studio Modeling Environment, Release 3.5.

[20] Yan Y., Tao H., He J., Huang S.Y. (2020). The HDOCK server for integrated protein–protein docking. Nat. Protoc..

[21] DeLano W.L. (2002). Pymol: An open-source molecular graphics tool. CCP4 Newsl. Protein Crystallogr..

[22] Ortega J.T., Jastrzebska B., Rangel H.R. (2022). Omicron SARS-CoV-2 Variant Spike Protein Shows an Increased Affinity to the Human ACE2 Receptor: An In Silico Analysis. Pathogens.

[23] Han P., Li L., Liu S., Wang Q., Zhang D., Xu Z., Han P., Li X., Peng Q., Su C. (2022). Receptor binding and complex structures of human ACE2 to spike RBD from Omicron and Delta SARS-CoV-2. Cell.

[24] Wu L., Zhou L., Mo M., Liu T., Wu C., Gong C., Lu K., Gong L., Zhu W., Xu Z. (2022). SARS-CoV-2 Omicron RBD shows weaker binding affinity than the currently dominant Delta variant to human ACE2. Signal Transduct. Target. Ther..

[25] Ackermann M., Verleden S.E., Kuehnel M., Haverich A., Welte T., Laenger F., Vanstapel A., Werlein C., Stark H., Tzankov A. (2020). Pulmonary Vascular Endothelialitis, Thrombosis, and Angiogenesis in COVID-19. N. Engl. J. Med..

[26] Davies J., Randeva H.S., Chatha K., Hall M., Spandidos D.A., Karteris E., Kyrou I. (2020). Neuropilin-1 as a new potential SARS-CoV-2 infection mediator implicated in the neurologic features and central nervous system involvement of COVID-19. Mol. Med. Rep..

[27] Kielian M. (2020). Enhancing host cell infection by SARS-CoV-2. Science.

[28] Lim K.H., Yang S., Kim S.H., Joo J.Y. (2021). Identifying New COVID-19 Receptor Neuropilin-1 in Severe Alzheimer’s Disease Patients Group Brain Using Genome-Wide Association Study Approach. Front. Genet..

[29] Sarabipour S., Gabhann F.M. (2021). Targeting neuropilins as a viable SARS-CoV-2 treatment. FEBS J..

[30] Mayi B.S., Leibowitz J.A., Woods A.T., Ammon K.A., Liu A.E., Raja A. (2021). The role of Neuropilin-1 in COVID-19. PLoS Pathog..

