# Supplementary material for: Conservation and Enhanced Binding of SARS-CoV-2 Omicron Spike Protein to Coreceptor Neuropilin-1 Predicted by Docking Analysis
Source: Infect Dis Rep. 2022 Mar 29;14(2):243–9. doi: 10.3390/idr14020029 (PMC9024780; doi:10.3390/idr14020029)
Supplement: Supplementary file 1 [file idr-14-00029-s001.zip › idr-1631801-supplementary.pdf]

Communication

Conservation and Enhanced Binding of SARS-CoV-2 Omicron Spike Protein to Coreceptor Neuropilin-1 Predicted by Docking Analysis

Piyush Baindara [1,*], Dinata Roy [2], Santi M. Mandal [3] and Adam G. Schrum [1]

[1] Departments of Molecular Microbiology & Immunology, Surgery, and Biomedical, Biological, & Chemical Engineering, School of Medicine, College of Engineering, University of Missouri, Columbia, MO 65211, USA; schruma@health.missouri.edu

[2] Department of Zoology, Mizoram University, Aizawl 796004, Mizoram, India; sdinaroym9@gmail.com

[3] Central Research Facility, Indian Institute of Technology Kharagpur, Kharagpur 721302, West Bengal, India; mandalsm@gmail.com

* Correspondence: pbaindara@health.missouri.edu

Table S1. Amino acid interaction analysis of docking complex between Nrp1 and S protein (CendR) of Wuhan and Omicron variant.

Name	Distance	Category	Types	From	From chemistry	To	To chemistry
				**Nrp1_spolybasic (Wuhan)**			
**A:ARG39:NH1 - A:GLU541:OE1**	2.84844	Hydrogen Bond;Electrostatic	Salt Bridge	A:ARG39:NH1	H-Donor	A:GLU541:OE1	H-Acceptor
**A:ARG42:NH1 - A:GLU550:OE1**	2.47242	Hydrogen Bond;Electrostatic	Salt Bridge	A:ARG42:NH1	H-Donor	A:GLU550:OE1	H-Acceptor
**A:ARG39:NH2 - A:GLU550:OE2**	4.5994	Electrostatic	Attractive Charge	A:ARG39:NH2	Positive	A:GLU550:OE2	Negative
**A:ARG40:NH2 - A:GLU541:OE2**	3.78555	Electrostatic	Attractive Charge	A:ARG40:NH2	Positive	A:GLU541:OE2	Negative
**A:THR35:N - A:CYS275:SG**	3.39395	Hydrogen Bond	Conventional Hydrogen Bond	A:THR35:N	H-Donor	A:CYS275:SG	H-Acceptor
**A:ARG39:NH2 - A:GLY542:O**	3.11931	Hydrogen Bond	Conventional Hydrogen Bond	A:ARG39:NH2	H-Donor	A:GLY542:O	H-Acceptor
**A:ALA41:N - A:PRO38:O**	3.10368	Hydrogen Bond	Conventional Hydrogen Bond	A:ALA41:N	H-Donor	A:PRO38:O	H-Acceptor
**A:ARG42:N - A:PRO38:O**	3.06475	Hydrogen Bond	Conventional Hydrogen Bond	A:ARG42:N	H-Donor	A:PRO38:O	H-Acceptor
**A:PRO398:CD - A:ARG42:O**	3.53223	Hydrogen Bond	Carbon Hydrogen Bond	A:PRO398:CD	H-Donor	A:ARG42:O	H-Acceptor
**A:PRO38:CD - A:THR553:OG1**	2.73466	Hydrogen Bond	Carbon Hydrogen Bond	A:PRO38:CD	H-Donor	A:THR553:OG1	H-Acceptor
**A:ARG42:CD - A:THR35:O**	3.18632	Hydrogen Bond	Carbon Hydrogen Bond	A:ARG42:CD	H-Donor	A:THR35:O	H-Acceptor
**A:ALA41 - A:PRO38**	4.14175	Hydrophobic	Alkyl	A:ALA41	Alkyl	A:PRO38	Alkyl
**A:ARG42 - A:PRO398**	4.30074	Hydrophobic	Alkyl	A:ARG42	Alkyl	A:PRO398	Alkyl
				**Nrp1_spolybasic (Omicron)**			
**A:ARG45:NH2 - A:GLU550:OE1**	3.18058	Hydrogen Bond;Electrostatic	Salt Bridge	A:ARG45:NH2	H-Donor	A:GLU550:OE1	H-Acceptor
**A:LYS41:NZ - A:GLU541:OE2**	5.05473	Electrostatic	Attractive Charge	A:LYS41:NZ	Positive	A:GLU541:OE2	Negative
**A:ARG45:NH1 - A:GLU550:OE2**	4.70486	Electrostatic	Attractive Charge	A:ARG45:NH1	Positive	A:GLU550:OE2	Negative
**A:ARG44:NH1 - A:CYS431:O**	2.88815	Hydrogen Bond	Conventional Hydrogen Bond	A:ARG44:NH1	H-Donor	A:CYS431:O	H-Acceptor
**A:ARG45:N - A:SER42:O**	3.03212	Hydrogen Bond	Conventional Hydrogen Bond	A:ARG45:N	H-Donor	A:SER42:O	H-Acceptor
**A:ARG45:NH2 - A:PRO398:O**	2.80779	Hydrogen Bond	Conventional Hydrogen Bond	A:ARG45:NH2	H-Donor	A:PRO398:O	H-Acceptor
**A:ARG45:CD - A:PRO398:O**	3.43562	Hydrogen Bond	Carbon Hydrogen Bond	A:ARG45:CD	H-Donor	A:PRO398:O	H-Acceptor
**A:ARG44 - A:LEU551**	4.58683	Hydrophobic	Alkyl	A:ARG44	Alkyl	A:LEU551	Alkyl
**A:ARG45 - A:PRO398**	3.73945	Hydrophobic	Alkyl	A:ARG45	Alkyl	A:PRO398	Alkyl
**A:ALA46 - A:CYS275**	4.50989	Hydrophobic	Alkyl	A:ALA46	Alkyl	A:CYS275	Alkyl
**A:ALA46 - A:PRO398**	4.72647	Hydrophobic	Alkyl	A:ALA46	Alkyl	A:PRO398	Alkyl
**A:PHE335 - A:ARG44**	5.13131	Hydrophobic	Pi-Alkyl	A:PHE335	Pi-Orbitals	A:ARG44	Alkyl
**A:PHE335 - A:ARG45**	4.39468	Hydrophobic	Pi-Alkyl	A:PHE335	Pi-Orbitals	A:ARG45	Alkyl
**A:TYR429 - A:ARG44**	4.3001	Hydrophobic	Pi-Alkyl	A:TYR429	Pi-Orbitals	A:ARG44	Alkyl
